# Supplementary figures and images for: Linkages between soil carbon, soil fertility and nitrogen fixation in Acacia senegal plantations of varying age in Sudan
Source: PeerJ. 2018 Jul 10;6:e5232. doi: 10.7717/peerj.5232 (PMC6044267; doi:10.7717/peerj.5232)

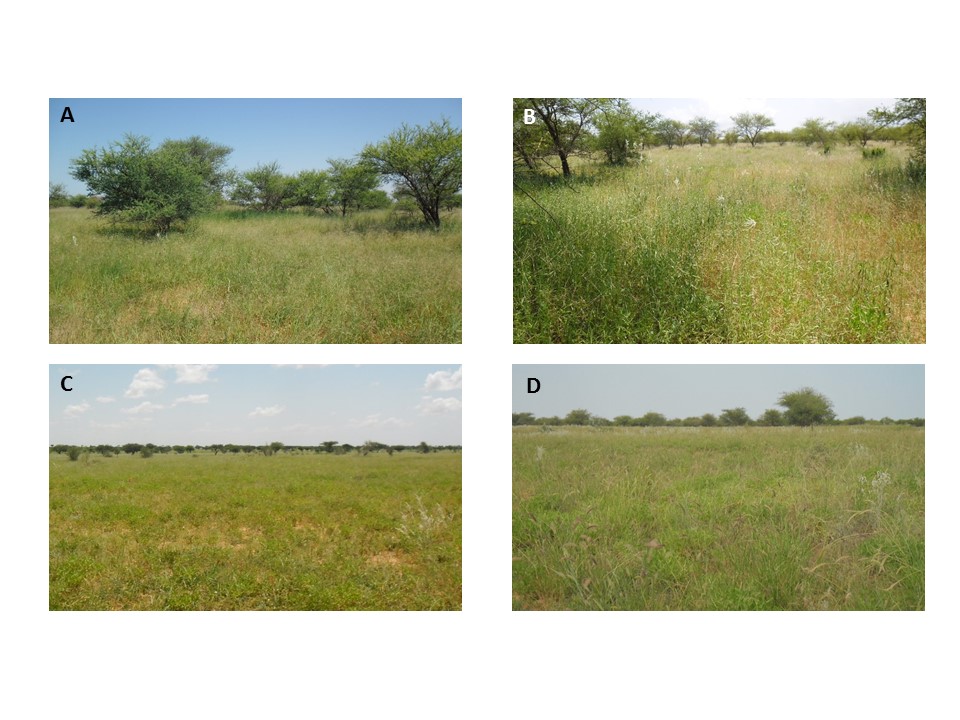

Supplement: Supplemental Information 1 — Photos taken by Wafa Abaker. [file peerj-06-5232-s001.jpg]
