# Supplementary material for: Linkages between soil carbon, soil fertility and nitrogen fixation in Acacia senegal plantations of varying age in Sudan
Source: PeerJ. 2018 Jul 10;6:e5232. doi: 10.7717/peerj.5232 (PMC6044267; doi:10.7717/peerj.5232)
Supplement: Supplemental Information 2 [file peerj-06-5232-s002.docx]

Supplementary material 2. List of ground vegetation species found at the two study sites.

| **Scientific name** | **Family name** | **Author** | **Type** | **Life form** |
| --- | --- | --- | --- | --- |
| *Cenchrus biflorus* | *Poaceae* | Roxb. | Grass | Annual |
| *Aristida funiculata* | *Poaceae* | Trin. & Rupr. | Grass | Annual |
| *Eragrostis tremula* | *Poaceae* | Hochst. ex Steud. | Grass | Annual |
| *Dactyloctenium aegyptium* | *Poaceae* | Willd. | Grass | Annual |
| *Echinochloa colona* | *Poaceae* | Link. | Grass | Annual |
| *Trianthema pentandra* | *Aizoaceae* | C. Jeffrey. | Herb | Annual |
| *Heliotropium supinum* | *Boraginaceae* | Linnaeus. | Herb | Annual |
| *Indigofera semitrijuga* | *Fabaceae* | Forssk. | Herb | Annual |
| *Justicia kotschyi* | *Acanthaceae* | Hochst. | Herb | Annual |
| *Zornia glochidiata* | *Fabaceae* | Rchb. ex DC. | Herb | Annual |
| *Acanthus spp.* | *Acanthaceae* | - | Herb | Annual |
| *Geigeria alata* | *Asteraceae* | Benth. & Hook.f. ex Oliv. & Hiern. | Herb | Annual |
