# Supplementary material for: Linkages between soil carbon, soil fertility and nitrogen fixation in Acacia senegal plantations of varying age in Sudan
Source: PeerJ. 2018 Jul 10;6:e5232. doi: 10.7717/peerj.5232 (PMC6044267; doi:10.7717/peerj.5232)
Supplement: Supplemental Information 3 — * grassland Values are mean values (n = 3) followed by standard deviation (in parentheses). [file peerj-06-5232-s003.docx]

**Supplementary material 3.** Soil stocks (g m^-2^; 0-50 cm layer) calculated using the minimum equivalent soil mass (ESM) basis (Lee et al. 2009) of SOC, N, total P, available P, total K and extractable K for grassland and plantations (under canopy) by age for the two study sites.

| Site | Age | SOC | N | P | P_av_ | K | K_ex_ |
| --- | --- | --- | --- | --- | --- | --- | --- |
| El Demokeya | 0* | 955 (51) | 106 (11) | 28.4 (3.2) | 2.2 (0.1) | 317 (35) | 38.2 (8.0) |
|  | 15 | 1032 (143) | 94 (10) | 35.5 (1.4) | 2.2 (0.1) | 292 (5) | 43.2 (4.6) |
|  | 24 | 1273 (117) | 154 (14) | 41.3 (7.4) | 2.2 (0.1) | 275 (29) | 52.0 (2.4) |
|  |  |  |  |  |  |  |  |
| El Hemaira | 0* | 892 (60) | 95 (1) | 27.6 (1.9) | 2.1 (0.6) | 349 (44) | 34.0 (0.6) |
|  | 7 | 1010 (193) | 92 (13) | 32.5 (1.1) | 2.1 (0.3) | 237(41) | 41.8 (3.0) |
|  | 15 | 1289 (186) | 140 (29) | 33.6 (6.7) | 2.1 (0.2) | 333 (64) | 51.6 (2.2) |
|  | 20 | 1458 (249) | 156 (34) | 35.3 (6.6) | 2.3 (0.3) | 360 (124) | 49.5 (10.0) |

* grassland

Values are mean values (n=3) followed by standard deviation (in parentheses).
